# 636. Meningococcal Vaccination and Antibiotic Prophylaxis Practices in Solid Organ Transplant Recipients undergoing Eculizumab Therapy

**DOI:** 10.1093/ofid/ofac492.688

**Published:** 2022-12-15

**Authors:** Eric Bhaimia, Zachary A Yetmar, Maryam Mahmood, Bernard Stacy, Jenise Stephen, Holenarasipur R Vikram, Lisa M Brumble

**Affiliations:** Rush University Medical Center, Rochester, MN; Mayo Clinic, Rochester, Minnesota; Mayo Clinic, Rochester, Minnesota; Mayo Clinic, Rochester, Minnesota; Mayo Clinic Arizona, Phoenix, Arizona; Mayo Clinic, Rochester, Minnesota; Mayo Clinic, Rochester, Minnesota

## Abstract

**Background:**

Eculizumab, an inhibitor of terminal complement activation, has been utilized in pre-transplant desensitization, treatment of antibody-mediated rejection and thrombotic microangiopathy in solid organ transplant recipients (SOTR). Eculizumab is associated with meningococcal disease, leading to guidelines for vaccination against serogroups ACWY (menACWY) and B (menB) at least 2 weeks prior to eculizumab therapy. In persons requiring emergent use of eculizumab, prophylaxis for at least 2 weeks is recommended. SOTRs respond suboptimally to vaccination, raising consideration for pre-transplant vaccination and prolonged prophylaxis in those undergoing eculizumab therapy. We sought to describe meningococcal vaccination and prophylaxis practices among SOTRs receiving eculizumab.

**Methods:**

We performed a retrospective chart review of meningococcal vaccination, including number and timing pre- or post-transplant, along with prophylaxis prescribing and duration in SOTRs receiving eculizumab across 3 distinct Mayo Clinic Enterprise sites (Rochester, Arizona, and Florida).

**Results:**

200 SOTRs receiving at least one dose of eculizumab between 1/1/2008 and 9/16/2021 were reviewed. Eculizumab indications included antibody mediated rejection (38%), desensitization (26%), and thrombotic microangiopathy (19%). 83% (166/200) of SOTRs received eculizumab for an unanticipated post-transplant complication. Vaccination rates were 82% (164/200) and 35.5% (71/200) for menACWY dose 1 and 2, respectively, and 54% (108/200) and 28.5% (57/200) for menB dose 1 and 2, respectively. The majority received their first vaccination post-transplant: menACWY 66.5% (133/200) and menB 2.5% (185/200). Among those without vaccination pre-eculizumab, 22.3% (41/184) were not prescribed prophylaxis. The median duration of prophylaxis was 62 (range 4-2651) days.

**Conclusion:**

Most SOTRs underwent meningococcal vaccinations near the time of eculizumab administration post-transplant for an unanticipated complication. One-fifth of those meeting criteria were not provided meningococcal prophylaxis. These findings may support pre-transplant administration of meningococcal vaccinations and heightened awareness for meningococcal prophylaxis in SOTRs receiving eculizumab.

**Disclosures:**

**All Authors**: No reported disclosures.

